# Small Pox

**Published:** 1868-12

**Authors:** 


### SMALL POX.

From extended and close observation, the following general deductions seem to be warranted:

1. Infantile vaccination is an almost perfect safeguard, until the fourteenth year.

2. At the beginning of *Fourteen*, the system gradually loses its capability of resistance, until about twenty-one, when many persons become almost as liable to small-pox, as if they had not been vaccinated.

3. This liability remains in full force until about forty-two, when the susceptibility begins to decline and continues for seven years to grow less and less, becoming extinct at about fifty, the period of life, when the general revolution of the body begins to take place, during which the system yields to decay, or takes a new lease of life, for two or three terms, of seven years each.

4. The great practical use to be made of these statements is—Let every youth be re-vaccinated on entering Fourteen. Let several attempts be made, so as to be certain of safety. As the malady is more liable to prevail in cities during winter, special attention is invited to the subject at this time.

